# Age-specific prevalence of the different clinical presentations of AD and FTD in young-onset dementia

**DOI:** 10.1007/s00415-024-12364-7

**Published:** 2024-04-21

**Authors:** Giovanna Zamboni, Riccardo Maramotti, Simone Salemme, Manuela Tondelli, Giorgia Adani, Giulia Vinceti, Chiara Carbone, Tommaso Filippini, Marco Vinceti, Giuseppe Pagnoni, Annalisa Chiari

**Affiliations:** 1 Dipartimento di Scienze Biomediche, Metaboliche e Neuroscienze, Università di Modena e Reggio Emilia, Via Giardini 1355, 41126 Modena, Italy; 2 Neurologia, Azienda Ospedaliero Universitaria di Modena, Modena, Italy; 3 Dipartimento di Scienze Fisiche, Informatiche e Matematiche, Università di Modena e Reggio Emilia, Modena, Italy

**Keywords:** Alzheimer’s disease, Frontotemporal dementia, Young-onset dementia, Early-onset dementia, Age-specific prevalence, Amnestic AD, Atypical AD, Posterior cortical atrophy, Logopenic variant of primary progressive aphasia, Aphasic FTD, Behavioural variant of FTD

## Abstract

**Background:**

Studies have shown that the prevalence of all-variants Alzheimer’s disease (AD) and frontotemporal dementia (FTD) both increase with age, even before the age of 65. However, it is not known whether their different clinical presentations all increase in prevalence with age in the same way.

**Methods:**

We studied the prevalence of the different clinical presentations of young-onset AD and FTD by 5-year age groups in a population-based study identifying all dementia patients with a diagnosis of AD and FTD and symptoms onset before age 65 in the Modena province, Italy. By using regression models of cumulative occurrences, we also estimated *age-specific prevalence* and compared the growth curves of the clinical presentations.

**Results:**

The prevalence of all-variants AD increased with age, from 18/1,000,000 in the 40–44 age group to 1411/1,000,000 in the 60–64 age group. The prevalence of all-variants FTD also increased with age, from 18/1,000,000 to 866/1,000,000. An estimation of *age-specific prevalence* functions of each clinical presentation showed that atypical non-amnestic AD and aphasic FTD grew the most in early ages, followed by the behavioural variant of FTD (bvFTD). Then, around the age of 60, amnestic AD took over and its *age-specific prevalence* continued to increase disproportionally compared to all the other clinical variants of AD and FTD, which, instead, started to decrease in prevalence.

**Conclusions:**

Amnestic AD is the clinical presentation that increases the most with advancing age, followed by bvFTD, suggesting that there is a differential vulnerability to the effect of ageing within the same neurodegenerative disease.

**Supplementary Information:**

The online version contains supplementary material available at 10.1007/s00415-024-12364-7.

## Introduction

The prevalence of all-cause dementia increases exponentially from ages 65 to 85, doubling every 5 years [1, 2], and continues to increase even after the age of 90 [3]. In a recent epidemiological study conducted in the province of Modena, Northern Italy, we showed that the prevalence of all-cause dementia increases exponentially with age also before the age of 65. More precisely, it increased from 74/1,000,000 in the 40–44 age group, to 508/1,000,000 in the 50–54 age group, to 2964/1,000,000 in the 60–64 age group [4].

Of all the causes of dementia, Alzheimer’s disease (AD) is certainly the most common one, accounting for 70% of the cases of late-onset dementia [5]. It has been recently shown that it is also the most common cause of young-onset dementia (YOD), i.e. dementia with symptoms occurring before the age of 65 [4, 6, 7], although older studies had found similar prevalences of AD and frontotemporal dementia (FTD) in the 45–64 years group [8]. It is established that the prevalence of AD increases with age across the entire age spectrum [9, 10] including ages before 65 [6, 11, 12]. However, it is not known whether the age-related increase of the prevalence of AD concerns only the most frequent, amnestic presentation, or also affects atypical presentations (also indicated as clinical variants, syndromes, or phenotypes) such as the logopenic variant of primary progressive aphasia (lvPPA), posterior cortical atrophy (PCA) and behavioural-dysexecutive variant (be-dAD). While studies from clinical cohorts suggest that these atypical presentations of AD usually occur in patients in their sixth and seventh decade [13, 14] and that the relative proportion of patients with atypical presentations is lower in older than in younger populations [15, 16], it has been suggested that the absolute number of cases with atypical presentations may still be higher in the older than in the younger populations, given the large number of people with AD in the former [17].

The second most prevalent form of YOD is frontotemporal dementia (FTD) [4, 6]. Although some epidemiological studies focussing on YOD have reported that the prevalence of FTD peaks between 55 and 59 years of age [6, 7, 11, 18], other studies have suggested that the prevalence of FTD increases with age [12, 19], although evidence supporting a continuous age-related increase of FTD is not as strong as that which exists for AD. Recent studies reporting the epidemiology of the whole frontotemporal lobar degeneration (FTLD) spectrum (i.e., including FTD as well as motor syndromes) across all the ages have reported that prevalence peaks at 65–69 years of age [20] and incidence peaks at the age of 71 [21] or even later, at 75–79 years of age [22]. Accordingly, a systematic review of the epidemiology of FTD did not show a lower FTD prevalence or incidence amongst older subjects compared to individuals younger than 65 [23]. Like AD, it is not known whether the clinical presentations of FTD increase with age in the same way, or the age-related increase is only driven by its most frequent presentation.

We aimed at studying the prevalence by age of onset of the different presentations of young-onset AD (including its amnestic and atypical presentations) and FTD (including its behavioural and aphasic presentations) with a population-based study. We hypothesised that demonstrating that the typical, amnestic presentation of AD is the only presentation of dementia that truly increases in prevalence with age would suggest that there are biological reasons that make it especially vulnerable to the effect of ageing. Showing that the prevalence of the atypical presentations of AD as well as of FTD also increases with age would instead suggest that different presentations as well as different neurodegenerative diseases are equally vulnerable to the effect of ageing, and therefore, there is no specific interaction between ageing and their distinguishing biological factors. Focussing on YOD allowed us to have more comparable numbers between different clinical presentations. In addition, in YOD, co-pathology occurs less frequently than in older-onset dementia, therefore the classification of different clinical presentations is more clearly biomarker—and thus biologically—driven and the comparisons are cleaner [24].

## Methods

Prevalent young-onset AD and FTD cases were identified in a population study conducted in the province of Modena, Northern Italy [4], involving all the residents alive on census day (June 30th, 2019) who had received a diagnosis of YOD. For FTD we purposefully focussed on the three core clinical syndromes [25], including the most common behavioural variant of FTD (bvFTD) and the two language syndromes, namely, nonfluent/agrammatic variant of primary progressive aphasia (nfvPPA) and semantic variant of primary progressive aphasia (svPPA). We did not include corticobasal syndrome and progressive supranuclear palsy, which together with FTD are part of the pathological concept of FTLD, as the number of cases with these two syndromes was too low to estimate their prevalence by age of onset.

Patient ascertainment involved the extended network of dementia services existing in the province of Modena, which includes inpatient and outpatient neurology, psychiatry and geriatric clinics covering the entire province and is part of the Italian National Health System (Sistema Sanitario Nazionale). The diagnosis of the clinical presentation was established by expert cognitive neurologists (A.C., G.V., M.T., and G.Z.) through the use of most recent diagnostic criteria for AD [26], its behavioural-dysexecutive variant (be-dAD) [27], bvFTD [28], primary progressive aphasia (PPA) and its variants [29], and PCA [13]. Exclusion criteria were co-existing diagnoses of developmental disorders, longstanding history of schizophrenia and bipolar disorder, cognitive impairment in the context of a neurological disorder in which non-cognitive symptoms were the most disabling, age younger than 30, and residence outside the province of Modena on census day. Diagnoses were based on neurological examination, clinical history, neuropsychological assessment, and brain magnetic resonance imaging (MRI) and also supported, when indicated, by measurement of cerebrospinal fluid (CSF) and amyloid PET imaging biomarkers. Prevalence and incidence of young-onset AD and FTD have been reported in [4]. Here we study the *prevalence by age of onset* of the *different presentations* of young-onset AD and FTD.

The study was conducted in accordance with local clinical research regulations and conformed to the Declaration of Helsinki. All subjects provided informed written consent (Study Number 186/2016, approved by the ethical committee Area Vasta Emilia Nord, Italy).

Prevalent cases of each AD and FTD presentation were stratified according to 5-year age groups and prevalence rates computed. In addition, *age-specific prevalence* (i.e., the prevalence stratified according to 1-year age groups) was calculated using as denominator the Modena province residing population on January 1st, 2019. The *age-specific prevalence* function *P*(*x*) = {number of prevalent cases with symptoms onset at age *x*} tended to increase with advancing age but showed fluctuations because of the small age groups. Therefore, to obtain more robustness, *age-specific prevalence* was estimated through a regression model. For this purpose, the *cumulative occurrence* function *F*(*x*) = {number of cases with symptoms onset by age *x*} was considered. The *cumulative occurrence* function F is non-decreasing and relates to *age-specific prevalence* as follows:

$$P\left( x \right) = \left[ {F\left( x \right) - F\left( {x - 1} \right)} \right]/\left[ {{\text{number of residents of age }}x} \right]$$

It follows that estimating *cumulative occurrence* can also be useful to predict the *age-specific prevalence*. Fitting a function to a sample of points requires first a selection of the family of candidate functions. Growth rates that increase at the beginning and decline at the end can faithfully be described by non-linear models. The ideal curve model should accommodate the variations of the true curve with the smallest number of parameters [30]. The five-parameter Generalized Logistic function [31] provides a compromise between over-parameterized models that can fit data closely at the cost of a large variance in the predictions and under-parameterized models that suffer from large lack-of-fit error. The function incorporates the sigmoidal shape of the *cumulative occurrences* with respect to time:

$$F\left(x\right)\approx D+\frac{A-D}{{\left[1+{\left(\frac{x}{C}\right)}^{B}\right]}^{E}}$$

In our study, the independent variable x represents the age in the interval 30–64 years. The parameters *A*, *B*, *C*, *D*, *E* have the following meaning:

- *A* controls the inferior asymptote. In our data there are no cases for x < 42, thus *A* is fixed at zero.
- *B* refers to the steepness of the curve.
- *C* is related to the inflection point.
- *D* controls the superior asymptote.
- *E* is the asymmetry factor. When *E* = 1 the curve is symmetric around the inflection point.

Supplementary Fig. 1 shows the changes of the function’s shape for different parameter selections. Having fixed *A* = 0, a four-parameters regression was obtained. The best fit was selected by minimizing the residual sum of squares (RSS). The algorithm was implemented on MATLAB, using the function fit and adapting the LP5 script of Cardillo [32].The procedure was repeated several times, to find the best regression function for each of the following diagnostic groups: amnestic AD, atypical AD (lvPPA, PCA, and be-dAD), bvFTD, aphasic FTD (svPPA and nfPPA). A group including the three PPA (lvPPA, svPPA, nfPPA) was also separately considered. *Age-specific prevalence* of all-variants AD was calculated by summing up the estimates of *age-specific prevalence* of amnestic AD and atypical AD. *Age-specific prevalence* of FTD was calculated by summing up the estimates of *age-specific prevalence* of bvFTD and aphasic FTD.

## Results

We identified 172 patients with a diagnosis of young-onset AD or FTD on census day. The diagnosis of AD was supported by at least one biomarker of amyloidosis (either CSF, 69 patients, or Amyloid-PET, 6 patients) in 70.7% of cases. The diagnosis of FTD was supported by the absence of biomarkers of amyloidosis (either CSF, 35 patients, or Amyloid-PET, 5 patients) in 60.6% of cases.

**Fig. 1:**
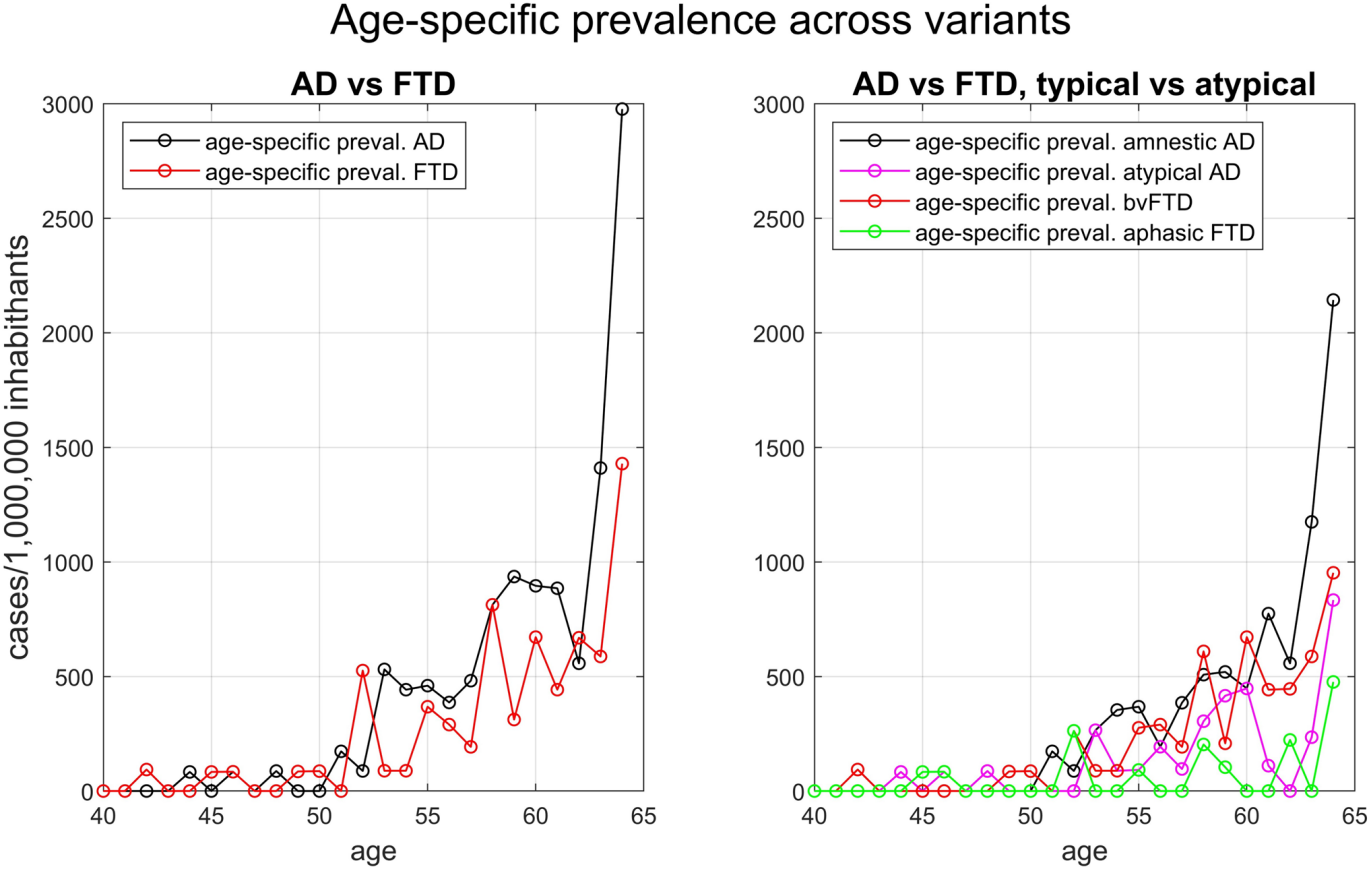
Age-specific prevalence across clinical presentations. The left panel shows all-variants AD and all-variants FTD. The right panel shows amnestic AD, atypical AD, behavioural FTD, and aphasic FTD

Table 1 reports the prevalence by 5-year range of age-of-onset of all-variants AD, all-variants FTD, and each of their clinical presentations. The prevalence of all-variants AD increased from 18/1,000,000 in the 40–44 age group to 1322/1,000,000 in the 60–64 age group. All-variants FTD increased from 18/1,000,000 in the 40–44 age group to 752/1,000,000 in the 60–64 age group. The prevalence by single year of age-of-onset is plotted in Fig. 1. Its oscillating behaviour justified the use of *cumulative occurrence* for fitting purposes.

**Table 1:** Crude prevalence of the different clinical presentations of young-onset AD and FTD at census date by 5-year range of age-of-onset per 1,000,000 inhabitants. Table also shows the total cases of AD and FTD and the number of residents in Modena province

Age of onset	Residents	AD										FTD							
		All AD		Amnestic AD		lvPPA		PCA		be-dAD		All FTD		bvFTD		svPPA		nfPPA	
		Total	Prev	Total	Prev	Total	Prev	Total	Prev	Total	Prev	Total	Prev	Total	Prev	Total	Prev	Total	Prev
40–44	54,241	1	18	0	0	0	0	1	19	0	0	1	18	1	18	0	0	0	0
45–49	59,048	2	34	0	0	2	34	0	0	0	0	3	51	1	17	2	34	0	0
50–54	57,072	14	245	10	175	2	35	2	35	0	0	9	158	6	105	1	18	2	35
55–59	51,060	31	607	20	392	5	98	1	20	5	98	20	392	16	313	2	39	2	39
60–64	43,860	58	1322	44	1003	10	228	4	91	0	0	33	752	27	616	6	137	0	0

**Fig. 2:**
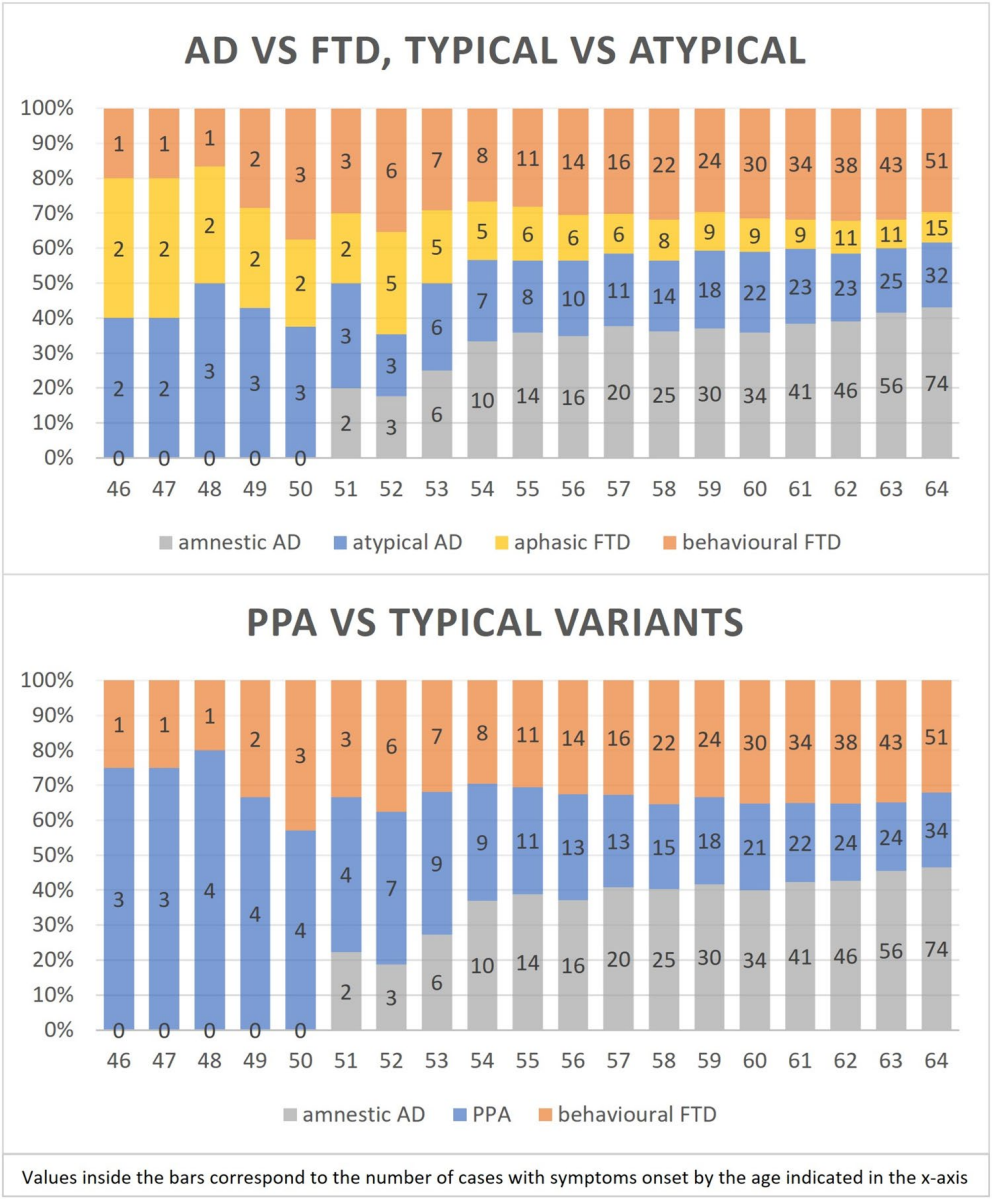
Percentage of *cumulative cases by year of symptoms onset* by clinical presentation. Numbers inside the bars indicate the number of cases that had developed symptoms by the age indicated in the x-axes. The top panel shows cumulative cases of amnestic AD, atypical AD, bvFTD, and aphasic FTD. In the bottom panel the lvPPA was grouped together with aphasic FTD (including svPPA and nfPPA) so that all PPA could be compared to amnestic AD and bvFTD

Figure 2 shows the percentage of cumulative cases by age of symptoms onset for the different variants of AD (grouped in amnestic and atypical) and FTD (grouped in behavioural and aphasic), as well as separately for all the PPA grouped together and compared to amnestic AD and bvFTD. All columns have the same height to highlight how the proportion of different clinical presentations changed with advancing age. More specifically, the graph shows that the relative proportion of cumulative cases of bvFTD remained stable across the ages, accounting for approximately 30% of total cases. On the other hand, the relative proportions of PPA and amnestic AD changed and balanced each other across the years. The proportion of PPA was highest before the age of 51 and started to decrease only after amnestic AD appeared and soon became more represented with the increase of age.

**Fig. 3:**
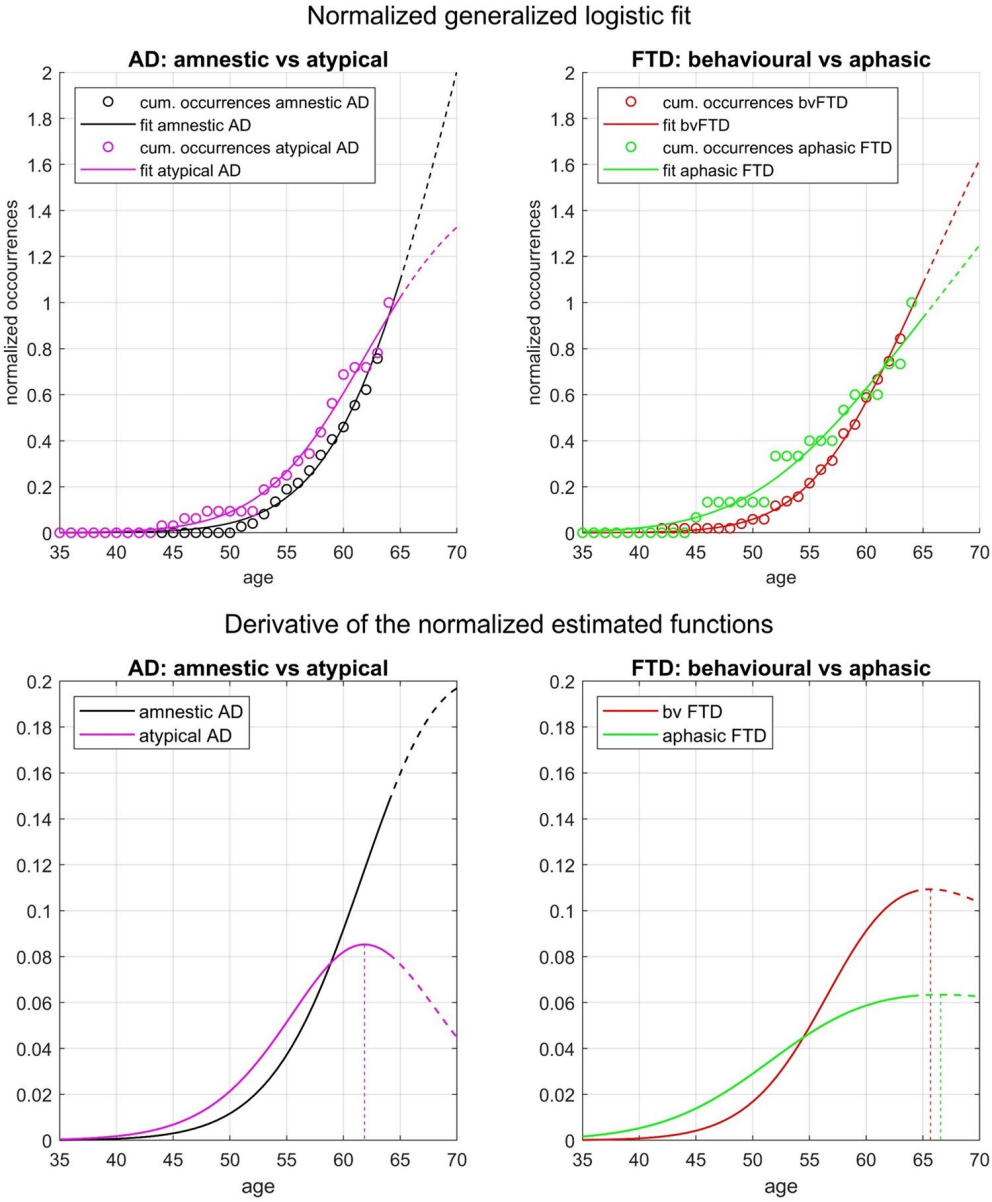
Top panels show the fit of the model. Points in the graphs indicate normalized cumulative occurrences, while the curves represent the estimation produced by the model. The top-left panel compares amnestic AD to atypical AD, while the top-right panel compares bvFTD to aphasic FTD. *Cumulative occurrences* at age 64 are scaled to 1, so that all normalized values fall in the 0–1 interval. Graphs are meant to compare the steepness of the curves, not the values on the *y*-axis, which were scaled to 1. Bottom panels indicate the first derivatives of the estimated functions. Note that the inflection point of a function corresponds to the maximum of its first derivative, thus the peaks, marked with vertical lines, can be interpreted as the age when the *cumulative occurrence* function slows its growth. For displaying purposes only, curves are extended beyond the age of 64 (dotted lines)

These observations were confirmed by the generalized logistic regression models (Supplementary Table 1). Indeed, the “worst” model explained 96.79% of the variability in the data (adjusted *R*^2^ = 0.9679). The *estimated cumulative occurrence functions* and their derivatives are represented in Fig. 3. To compare the growth rate (i.e., the slope of the curve) of different clinical presentations, the plotted values were scaled to the interval 0–1, so that *cumulative occurrences* at age 64 are set to 1 for all clinical presentations. The *normalized cumulative occurrence* of x is thus the percentage of people with a specific clinical presentation who had developed symptoms *by* age x, amongst those with that clinical presentation of YOD. Because the growth rate and the inflection point of a function are described by the value of its derivative and its maximum, respectively, the peaks of the graphs reported in the bottom part of Fig. 3 indicate the ages when *cumulative occurrence* functions slowed their growth and *age-specific prevalence* started to decrease. As for AD, the estimated function curve of amnestic AD never stopped increasing, whereas the estimated function curve of atypical AD started to decrease after age 62 (function inflection point). In younger ages, the estimated curve of atypical AD grew faster than amnestic AD, until the age of 59 (derivatives intersection at age 59), when the curve of amnestic AD started to grow much faster than that of atypical AD. As for FTD, the estimated function curve of both bvFTD and aphasic FTD continued to grow across all the considered age interval (i.e., their estimated functions inflection points fell right after 65). The curve of aphasic FTD grew faster than that of bvFTD until the age of 54 (aphasic FTD and bvFTD derivatives intersection), when bvFTD started to grow faster. Also, the curve of bvFTD grew faster than amnestic AD in younger ages until the age of 60 (amnestic AD and bvFTD derivatives intersection, not shown), when amnestic AD took over.

**Fig. 4:**
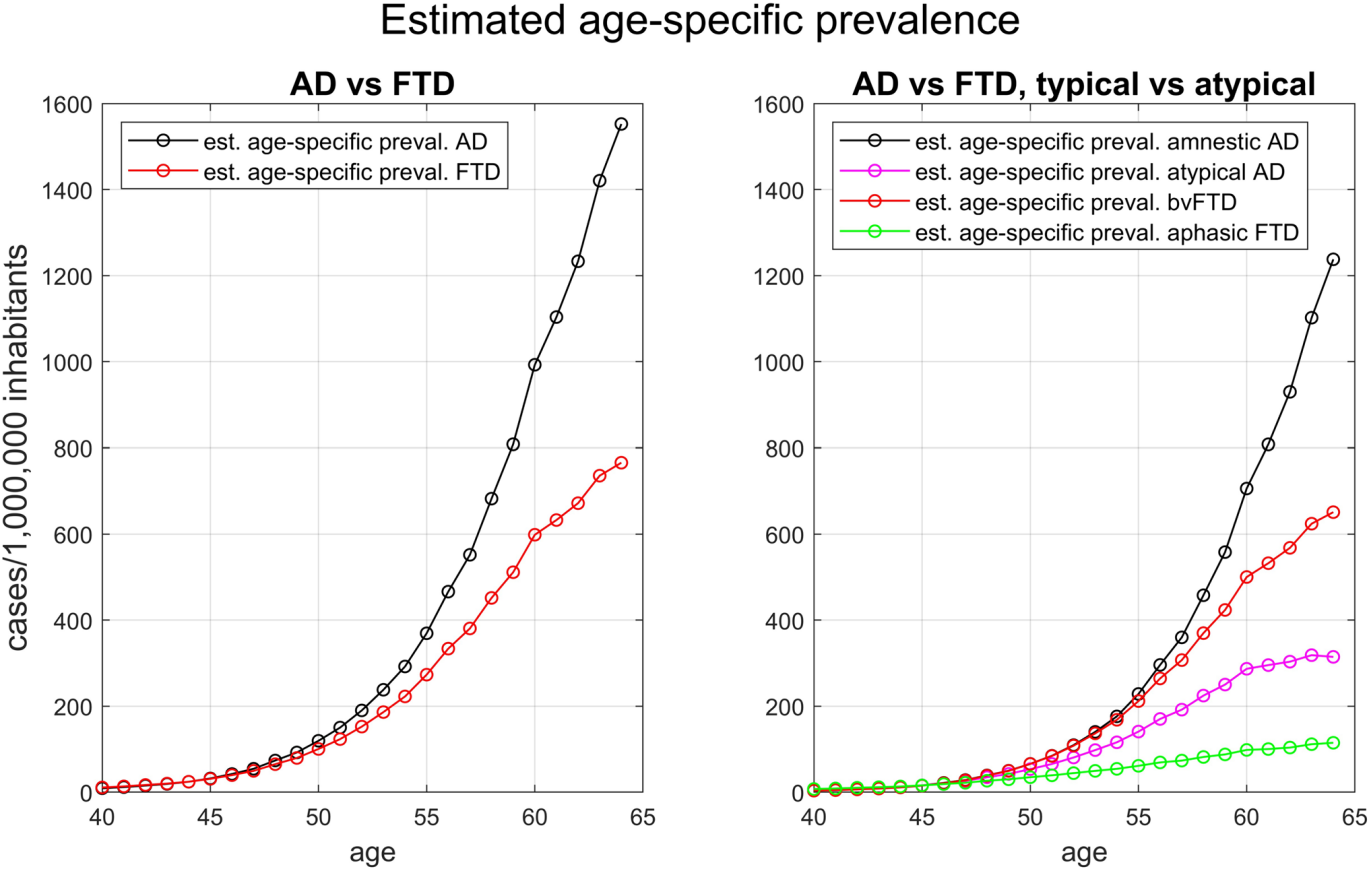
Estimated age-specific prevalence functions. The left panel compares all-variants AD versus all-variants FTD, while the right panel compares amnestic AD, atypical AD, bvFTD, and aphasic FTD

Figure 4 and Supplementary Table 2 show the *estimated age-specific prevalence* by age-of-onset obtained using the regression models of *cumulative occurrences* for all-variants AD and FTD and for their different clinical presentations. The slope of *estimated age-specific prevalence* function increased with advancing age in both diseases in early years, although the two curves started to separate after 50 years of age, with AD increasing much more than FTD. The difference was driven by amnestic AD: indeed, the *estimated age-specific prevalence* functions of all the clinical presentations increased with advancing age in early ages, but the slope of such increase started to flatten after the age of 60 for all the clinical presentations except for amnestic AD, which, by the age of 60, had overcome the growth rate of all the other presentations. Atypical AD and aphasic FTD (and consequently PPA) were the clinical presentations for which the *estimated age-specific prevalence* slowed the most after 60 (Supplementary Table 2). More precisely, the delta for *age-specific prevalence* between age 60 and age 64 was + 75.3% for amnestic AD (from 706 cases/1.000.000 to 1238/1,000,000), + 30.2% for bvFTD (from 500/1,000,000 to 651/1,000,000), + 17.3% for aphasic FTD (from 98/1,000,000 to 116/1,000,000), and + 9.8% for atypical AD (from 287/1,000,000 to 315/1,000,000).

## Discussion

The present study was aimed at establishing if the prevalence of the different clinical presentations of young-onset AD and FTD increases in the same way with ageing. We studied the break-down of prevalence by 5-year range of age-of-onset of the different clinical presentations of young-onset AD and FTD in a population-based study. We then estimated *age-specific prevalence*, i.e., prevalence stratified by single year of age-of-onset, with regression analyses to increase robustness and overcome limitations related to the fluctuations observed with small case numbers when counting prevalence figures.

The prevalence of all-variants AD increased by about 70 times from the 40–44 age group (18/1,000,000) to the 60–64 age group (1322/1,000,000). All-variants FTD increased by about 40 times from the 40–44 age group (18/1,000,000) to the 60–64 age group (752/1,000,000). The differences between AD and FTD were also clearly shown by their *estimated age-specific prevalence* (i.e., prevalence by year of age obtained from the estimation of regression models of *cumulative occurrences*), which were almost overlapping in the fifth decade, progressively separated in the sixth decade, and showed a sharp increase of AD in the first half of the seventh decade.

When looking at the different clinical presentations of AD and FTD, we found that the amnestic variant of AD increased disproportionally in *age-specific prevalence* compared to atypical AD (including PCA, lvPPA, and be-dAD) and all the presentations of FTD. Atypical AD and aphasic FTD (and therefore also the three PPA grouped together) were the clinical presentations for which the *estimated age-specific prevalence* grew the least and almost stabilized after the age of 60, whereas bvFTD remained the closest to amnestic AD, although the curve of its *age-specific prevalence* also started to flatten after the age of 60. This meant that as the overall prevalence of dementia increased with advancing age, the proportion of PPA decreased giving way to the sharp increase in the proportion of amnestic AD, while the proportion of bvFTD remained stable across the examined age interval.

The growth rates and the ages when growth curves slowed were compared using the derivative of the *age-specific prevalence* function. The derivative of atypical AD curve peaked at age 62, indicating that the *age-specific prevalence* started to decrease at that age. The derivatives of both bvFTD and aphasic FTD peaked just after age 65, while the growth curve of amnestic AD never stopped or stabilized.

These results suggest that the most frequent clinical presentation of AD and FTD (amnestic AD and bvFTD) are also those whose prevalence increases the most with advancing age. While age-related increase slows for bvFTD between ages 60 and 65, it accelerates for amnestic AD, which increases disproportionally compared to all the other clinical presentations of AD and FTD.

The specific effect of ageing on the amnestic variant of AD cannot be explained in terms of underlying pathology, which is shared by the atypical variants of AD. We speculate that it rather relates to the fact that the vulnerability of medial temporal structures to disease manifest itself only if no other pre-existing/developmental vulnerabilities have already taken their toll on the clinical presentation: on the one hand, there is increasing evidence that some neurodevelopmental conditions are associated with neurodegenerative diseases. More specifically, dyslexia is overrepresented in patients with PPA [33], visuospatial learning disabilities in PCA [34], non-right handedness in svPPA [35]. This has suggested that atypical neurodevelopmental features confer added vulnerability to specific clinical presentations of adult-onset neurodegenerative diseases [33, 34]. On the other hand, normal ageing is associated with brain changes: the hippocampus undergoes volume loss especially after the age of 50 [36]. This is associated with decreased episodic memory in non-cognitively impaired individuals [37]. Interestingly, the hippocampal subregions more involved in age-related volume loss, i.e., cornu ammonis 1–4 and dentate gyrus, are also the most affected by AD pathology [38]. Normal ageing also affects functioning and structure of the pre-frontal cortex [39, 40]. Changes related to atypical neurodevelopmental features are present in the brain since childhood, largely preceding the onset of neurodegenerative diseases, while age-related hippocampal atrophy and frontal lobe dysfunction represent age-specific modifications. If a neurodegenerative disease occurs in the adult life, having had atypical neurodevelopmental features may therefore add a vulnerability to presentations associated with brain areas different from the ones mostly involved in normal ageing. We speculate that this *added* vulnerability may explain the increase in prevalence in early ages of atypical and aphasic clinical presentations. Vice-versa, if the same neurodegenerative disease occurs in the absence of such pre-existing added vulnerability, the effect of ageing alone may contribute more substantially to the clinical presentation. The disease will, therefore, manifest with presentations associated with the involvement of brain structures primarily exposed to age-related modifications. These clinical presentations may therefore increase in prevalence exponentially with ageing. Further in-depth analysis of patients’ genetic profiles and developmental brain features across the clinical presentations of neurodegenerative dementias are needed to clarify these hypotheses and to better understand their specific risk factors.

The present study has limitations. First, the number of prevalent cases was low and may have biased the identification of associations between age and the less frequent presentations of AD or FTD. However, we deem this to be unlikely, as the atypical presentations of AD and aphasic presentations of FTD were pooled together to increase group sample size and the confidence of the estimates. Second, the regression analysis was performed on crude occurrences. The decision not to stratify by sex was based on the need to avoid reducing the model’s statistical power. Furthermore, there is no consensus on whether YOD is differentially frequent between sexes [41]. Third, neuropathological confirmation of the diagnoses was not available. However, differently from the majority of existing literature on the epidemiology of dementia, in which the lack of information on amyloid status may have favoured overestimation of AD and underestimation of FTD, in the present study the diagnosis of AD and FTD was supported by the amyloidosis biomarker in most cases.

In conclusion, to the best of our knowledge, this is the first report of the *age-specific prevalence* of the different clinical presentations of AD and FTD in YOD. These presentations behave differently as age increases, with the proportion of the atypical variants of AD and the aphasic variants of FTD being gradually “compressed” as the proportion of bvFTD and, especially, amnestic AD, increases. This suggests that a differential vulnerability of the different presentations to the effect of ageing may contribute to phenotypical variability within the same neurodegenerative disease.

### Supplementary Information

Below is the link to the electronic supplementary material.

Supplementary file1 (DOCX 124 KB)

## Data Availability

Data not provided in the article because of space limitations may be shared (anonymized) at the request of any qualified investigator for purposes of replicating procedures and results upon permission by the ethics committee.
