# Supplementary material for: Age-specific prevalence of the different clinical presentations of AD and FTD in young-onset dementia
Source: J Neurol. 2024 Apr 21;271(7):4326–35. doi: 10.1007/s00415-024-12364-7 (PMC11233291; doi:10.1007/s00415-024-12364-7)
Supplement: Supplementary file 1 — Supplementary file1 (DOCX 124 KB) [file 415_2024_12364_MOESM1_ESM.docx]

**Supplementary Figure 1** Effect of varying parameters on generalized logistic function, showing its adaptability to differently-shaped potential time courses of cumulative occurrence

**
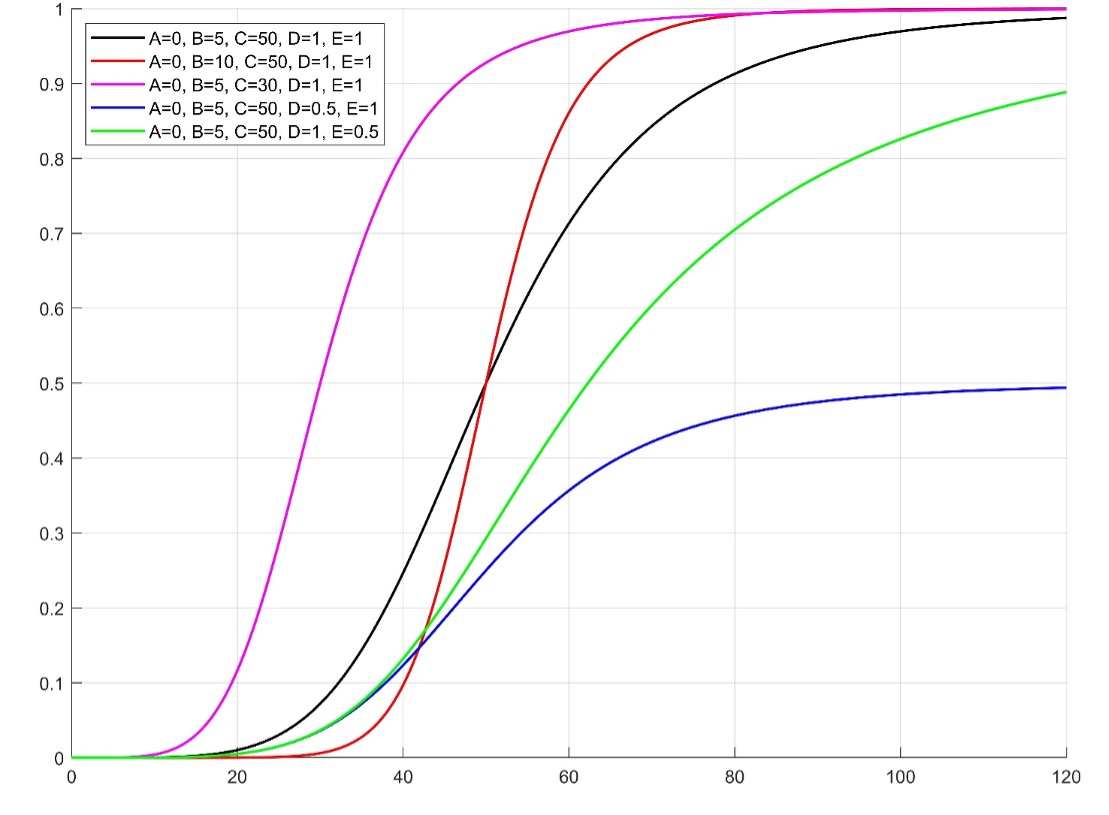
**

**Supplementary Tables**

**Supplementary Table 1**. R-squared and adjusted R-squared of the generalized logistic regression models.

|  | amnestic AD | atypical AD | bvFTD | aphasic FTD | PPA |
| --- | --- | --- | --- | --- | --- |
| R-squared | 0.9911 | 0.9878 | 0.9980 | 0.9701 | 0.9832 |
| Adjusted R-square | 0.9900 | 0.9863 | 0.9978 | 0.9679 | 0.9813 |

**Supplementary Table 2**. Estimated age-specific prevalence of the different clinical presentation of young onset AD and FTD. Values are expressed in units of cases per 1 000 000 inhabitants.

| **Age** | **all AD** | amnestic AD | atypical AD | **all FTD** | bvFTD | aphasic FTD | PPA |
| --- | --- | --- | --- | --- | --- | --- | --- |
| **40** | 9 | 4 | 5 | 11 | 4 | 7 | 12 |
| **41** | 12 | 5 | 6 | 14 | 5 | 9 | 14 |
| **42** | 15 | 7 | 8 | 17 | 7 | 10 | 17 |
| **43** | 19 | 9 | 10 | 20 | 8 | 12 | 19 |
| **44** | 24 | 12 | 12 | 24 | 11 | 13 | 23 |
| **45** | 32 | 16 | 16 | 31 | 15 | 16 | 27 |
| **46** | 42 | 22 | 21 | 40 | 21 | 19 | 33 |
| **47** | 55 | 28 | 26 | 50 | 28 | 22 | 39 |
| **48** | 74 | 39 | 35 | 65 | 38 | 27 | 48 |
| **49** | 92 | 50 | 42 | 80 | 50 | 30 | 55 |
| **50** | 120 | 66 | 54 | 100 | 66 | 35 | 66 |
| **51** | 150 | 85 | 66 | 123 | 84 | 39 | 76 |
| **52** | 190 | 109 | 81 | 152 | 108 | 44 | 88 |
| **53** | 238 | 140 | 98 | 186 | 137 | 50 | 102 |
| **54** | 292 | 176 | 116 | 223 | 168 | 54 | 115 |
| **55** | 369 | 228 | 141 | 273 | 212 | 61 | 134 |
| **56** | 466 | 296 | 170 | 334 | 264 | 69 | 156 |
| **57** | 552 | 360 | 192 | 381 | 307 | 73 | 172 |
| **58** | 682 | 458 | 225 | 452 | 370 | 82 | 198 |
| **59** | 808 | 558 | 250 | 511 | 424 | 88 | 220 |
| **60** | 993 | 706 | 287 | 598 | 500 | 98 | 254 |
| **61** | 1104 | 808 | 295 | 633 | 532 | 100 | 267 |
| **62** | 1233 | 930 | 303 | 672 | 568 | 104 | 285 |
| **63** | 1421 | 1102 | 319 | 736 | 624 | 112 | 315 |
| **64** | 1552 | 1238 | 315 | 766 | 651 | 115 | 332 |
